# Metagenomic analysis of microbiological changes on the ocular surface of diabetic children and adolescents with a dry eye

**DOI:** 10.1186/s12866-023-03013-6

**Published:** 2023-10-06

**Authors:** Zhangling Chen, Ying Xiao, Yan Jia, Qiurong Lin, Yu Qian, Lipu Cui, Zhaoyu Xiang, Mingfang Li, Chenhao Yang, Haidong Zou

**Affiliations:** 1 Department of Ophthalmology, Shanghai General Hospital, Nanjing Medical University, Shanghai, China; 2 Department of Ophthalmology, Shanghai Songjiang District Central Hospital, Shanghai, China; 3 Department of Ophthalmology, Children’s Hospital of Fudan University, Shanghai, China; 4 Shanghai Eye Diseases Prevention & Treatment Center/Shanghai Eye Hospital, Shanghai, China; 5 Department of Ophthalmology, Shanghai General Hospital, School of Medicine, Shanghai Jiao Tong University, Shanghai, China; 6 Shanghai Key Laboratory of Fundus Diseases, Shanghai, China; 7 National Clinical Research Center for Eye Diseases, Shanghai, China; 8 Shanghai Engineering Center for Precise Diagnosis and Treatment of Eye Diseases, Shanghai, China

**Keywords:** Children and adolescents, Diabetes mellitus, Dry eye, Ocular surface, Microecology, Metagenome

## Abstract

**Background:**

Microbiome changes on the ocular surface may cause dry eyes. A metagenome assay was used to compare the microbiome composition and function of the ocular surface between diabetic children and adolescents with dry eye, diabetic children and adolescents without dry eye, and normal children.

**Materials and methods:**

Twenty children and adolescents aged 8 to 16 with diabetes were selected from the Shanghai Children and Adolescent Diabetes Eye Study. Ten healthy children and adolescents belonging to the same age group were selected from the outpatient clinic during the same period. The participants were classified into the dry eye group (DM-DE group, *n* = 10), the non-dry eye group (DM-NDE group, *n* = 10) and the normal group (NDM group, *n* = 10). A conjunctival sac swab was collected for metagenomic sequencing, and the relationship between the microbiome composition and functional gene differences on the ocular surface with dry eye was studied.

**Results:**

The classification composition and metabolic function of the microorganisms on the ocular surface of children in the 3 groups were analyzed. It was found that children’s ocular microbiota was composed of bacteria, viruses and fungi. There were significant differences in α diversity and β diversity of microbial composition of ocular surface between DM-DE group and NDM group(*P<*0.05). There were significant differences in α and β diversity of metabolic pathways between the two groups(*P<*0.05). The functional pathways of ocular surface microorganisms in diabetic children with dry eyes were mainly derived from human disease, antibiotic resistance genes, carbohydrate, coenzyme and lipid transport and metabolism-related functional genes; In normal children, the functional pathways were mainly derived from replication, recombination, repair, signal transduction and defense-related functional genes.

**Conclusion:**

The DM-DE group have unique microbial composition and functional metabolic pathways. The dominant species and unique metabolic pathways of the ocular surface in the DM-DE group may be involved in the pathogenesis of dry eye in diabetic children.

## Introduction

In recent years, due to changes in lifestyle and everyday habits that affect the eyes, the incidence of dry eye (DE) has been increasing every year, and more causes of DE have been identified. DE resulting from systemic diseases, especially type 1 and 2 diabetes mellitus (DM), has attracted an increasing amount of attention, and the prevalence of diabetic dry eye is higher than that of non-diabetic dry eye [1, 2]. In 2018, our research group found out during the cross-sectional study that the percentage of prevalence of dry eye in diabetic children was 28.95%, while in normal children it was only 5.00% [1]. Through a 3-year regular follow-up of these diabetic children, it was noted that the incidence of dry eye in diabetic children was 22.5% within the period, which meant that the annual average incidence was 7.5% [3]. The eye damage caused by dry eye in children with diabetes starts from the onset of the disease, lasts for a long while, and affects the quality of life. However, the pathogenesis of dry eye in children with diabetes is not clear at present, and the methods of treatment are unclear either.

Ocular surface microorganism (OSM) features gradually draw people’s attention in recent years [4, 5]. Studies showed that the changes of ocular surface *Staphylococcus aureus*, *Coagulase-negative staphylococcus* and *Corynebacterium* were related to the prevalence of DE [6, 7]. Other studies have found that OSM disorder is also involved in the development of DE [8–15]. The possible mechanism of the high incidence of DE and OSM disorder in DM population is that the high glucose environment activates immune cells and induces ocular surface inflammation by increasing the osmotic pressure of tears and promoting the formation of advanced glycation end products (AGEs), thus enhancing the ocular surface immune response to OSM and cause OSM disorder [16–18].

The development of next-generation sequencing technologies and bioinformatics tools can facilitate the acquisition of human microbiome characteristics [19]. Compared with traditional culture methods, metagenomic assays can not only identify the species composition of the ocular surface microbiome but also further analyze the functional genes. Shotgun metagenomic sequencing is an unamplified method used to characterize the complete catalog of microbial species and functional genes. This method has been widely used in human intestinal microbiome studies and gradually applied to eye microbiome studies [20, 21]. In this study, metagenomic detection was used to study the microbial species composition of the dry eye surface in diabetic children and the changes in functional genes that may be involved in the pathogenesis of dry eye. Furthermore, this method was used to analyze the microbial population composition of the dry eye surface in diabetic children and the relationship between functional genes and the pathogenesis of dry eye.

## Materials and methods (Fig. 1)

**Fig. 1:**
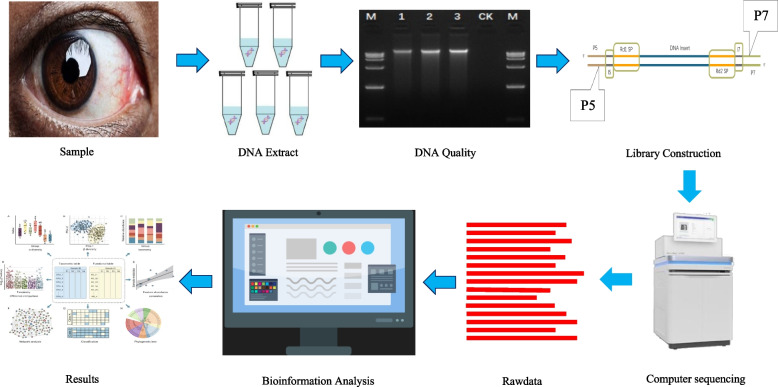
Methodology

### Study design and setting

In the SCADE study, 10 children and adolescents with diabetes who had DE (DM-DE group), 10 children and adolescents with diabetes who did not have DE (DM-NDE group), and 10 age-matched healthy controls without diabetes (NDM group) who visited the outpatient clinic in January 2022 were included in the study. As the prevalence rate of dry eye in healthy children is very low (i.e., only 5.00%) [1], it is difficult to collect a sufficient sample size of normal children with dry eye, so there was no group of normal children with dry eye in this study.

### Study inclusion and exclusion criteria

 The inclusion criteria for study participants were as follows: (1) both subjects and their guardians were fully informed of the study, and informed consent have been obtained from their legal guardians; (2) subjects aged 8–16 years; (3) subjects with diabetes were diagnosed with type 1 diabetes according to the diagnostic criteria of the World Health Organization (WHO) [22]; (4) the normal control group had no other systemic diseases or history of DE; and (5) all subjects were able to cooperate in completing the eye examination. The exclusion criteria were as follows: (1) other ocular and systemic diseases affecting the secretion and quality of tears, namely, eyelid diseases trichiasis, entropion, ectropion, ptosis, incomplete eyelid closure, etc.; (2) conjunctival diseases: acute and chronic infectious conjunctivitis, allergic conjunctivitis, conjunctival stones, etc.; (3) severe ocular chemical injury complications and ocular trauma history within 6 months; (4) ocular surgery history within 6 months; (5) severe ocular complications caused by diabetes: cataract, retinopathy, optic atrophy, etc.; (6) treatment with eye drops; and (7) use of contact lenses for more than 1 month.

According to the International Dry Eye Workshop II (DEWS II) criteria for DE examination and diagnosis [23], the criteria for diagnosing DE were as follows: patient complained of one of the subjective symptoms, such as ocular dryness, foreign body sensation, burning sensation, fatigue, discomfort, eye redness, vision fluctuation, and other subjective symptoms; ocular surface disease index (OSDI) ≥ 13 points; tear break-up time (BUT) ≤ 5 s or Schirmer I test ≤ 5 mm/5 min; and a positive fluorescein sodium corneal staining in case of DE-related symptoms, OSDI ≥ 13, BUT > 5 s and ≤ 10 s or Schirmer I test > 5 mm/5 min and ≤ 10 mm/5 min. According to the above criteria, dry eye can be diagnosed in only one eye or both eyes.

### Specimen collection

On the day before specimen collection, the teenagers and children had enough sleep; On the day of sample collection, the teenagers and children avoided washing their eyes with water. Samples were obtained from 8:00 a.m. − 10:00 a.m. at the same time. Before sampling, a saline swab was used to wipe the participants’ eyelid skin twice; subsequently, a conjunctival sac swab was used to collect ocular surface microorganisms from the eyes. The entire procedure was performed aseptically. The collected samples were quickly placed in a tube containing DNA protecting fluid (GeWei Bio-Tech, Shanghai, China), stored in a refrigerator at -20 °C (Haier, Qingdao, China) for cryopreservation, and then used for testing and DNA extraction.

All children were arranged for specimen collection at Shanghai Eye Disease Prevention and Treatment Center/Shanghai Eye Hospital. Eye specimens were collected by a trained ophthalmologist (Z.C.) to ensure the consistency of the results.

### Metagenome DNA extraction and shotgun sequencing

Total microbial genomic DNA samples were extracted using the OMEGA Mag-Bind Soil DNA Kit (M5635-02) (Omega Bio-Tek, Norcross, GA, USA), following the manufacturer’s instructions, and stored at -20 °C prior to further assessment. The quantity and quality of extracted DNAs were measured using a Qubit™ 4 Fluorometer, with WiFi: Q33238 (Qubit™ Assay Tubes: Q32856; Qubit™ 1X dsDNA HS Assay Kit: Q33231) (Invitrogen, USA) and agarose gel electrophoresis, respectively. The extracted microbial DNA was processed to construct metagenome shotgun sequencing libraries with insert sizes of 400 bp by using Illumina TruSeq Nano DNA LT Library Preparation Kit. Each library was sequenced by Illumina NovaSeq platform (Illumina, USA) with PE150 strategy at Personal Biotechnology Co., Ltd. (Shanghai, China).

### Sequencing, bioinformatics, and statistical analysis

Based on the Illumina NovaSeq/HiSeq high-throughput sequencing platform, using the whole genome shotgun (WGS) strategy, the extracted total microflora metagenomic DNA was used as an mRNA template to synthesize double-stranded cDNA that was randomly interrupted into short fragments, and cDNA libraries of appropriate length were constructed. The cDNA libraries were subjected to double-paired (2 × 150 bp PE) sequencing. A library was constructed for each sample, and 6G of original data was obtained. Quality screening was performed on the original data of double-ended sequences off the high-throughput sequencing machine to remove the target sequences and obtain clean datasets that could be used for downstream metagenomic analysis. Species annotation was carried out on high-quality datasets and nonredundant amino acid sequences to obtain species composition spectra at the fine level of species and below species, and species composition analysis, diversity analysis, difference analysis, etc. To compare the diversity of different samples, the composition spectrum of all samples at the species level was leveled at the lowest sequencing depth to correct the diversity difference caused by sequencing depth. The common and unique species of each group were analyzed. According to the composition spectrum of each group at the species level, R software was used to calculate the number of common and unique species, and the number of common and unique species of each group was intuitively presented by a Venn diagram. QIIME software was used to map 2D or 3D data from PCoA to obtain spatial distribution characteristics based on metagenomic species composition and to quantify the size of differences between groups. The high-quality sequences were corrected, spliced and assembled, and the metagenomic contig sequence set was constructed. After the redundancy was removed, gene prediction was carried out to obtain the nonredundant amino acid sequence set. Functional annotation of amino acid sequences was carried out using a variety of common databases to obtain the abundance spectrum of functional groups of each level, and functional composition analysis, diversity analysis, difference analysis, etc. were performed. QIIME software was used to obtain the relative abundance distribution table of each sample corresponding to each functional level of each database. R software was used to design a bar chart for the primary and secondary functional groups or metabolic pathways in each sample. By using R software and QIIME software, PCoA was carried out on the composition spectrum of the functional units of the samples, and the difference distribution characteristics of the samples were described by two-dimensional and three-dimensional images. QIIME software was used for ANOSIM analysis, and 999 displacement tests were performed to determine whether the differences between the groups were statistically significant. LEfSe analysis software was used to analyze the functional abundance table of the KEGG [24] and EggNOG databases, and the PLS-DA discriminant model was constructed according to the functional composition spectrum and sample grouping data. IBM SPSS Statistics 22 was used for statistical processing of the basic data of the research objects. Single-factor ANOVA was used for age comparison of the three groups of children, and the chi-square test was used for gender comparison. *P* < 0.05 was considered statistically significant.

## Results

### Basic information of the study subjects

The basic information of 30 children (20 diabetic children and 10 normal children) is shown in Table 1. The diabetic children with dry eye group (DM-DE), diabetic children without dry eye group (DM-NDE) and normal children group (NDM) were matched in terms of sex, age and BMI, and thus, there was no statistically significant difference (*P* > 0.05).

**Table 1:** Basic information of 30 children and adolescents

	group			*x*^*2*^*or F*	*P value*
	NDM	DM-NDE	DM-DE		
Gender(male/female)	6/4	4/6	5/5	*x*^*2*^ *= 0.69*	0.71
Age(years)	13.06 ± 2.33	12.96 ± 2.88	13.47 ± 2.64	* F = 0.13*	0.88
BMI (kg/m^2^)	20.45 ± 3.96	20.05 ± 2.66	21.17 ± 2.33	* F = 0.34*	0.72

*BMI* Body mass index

### Bioinformatics analysis

**Fig. 2:**
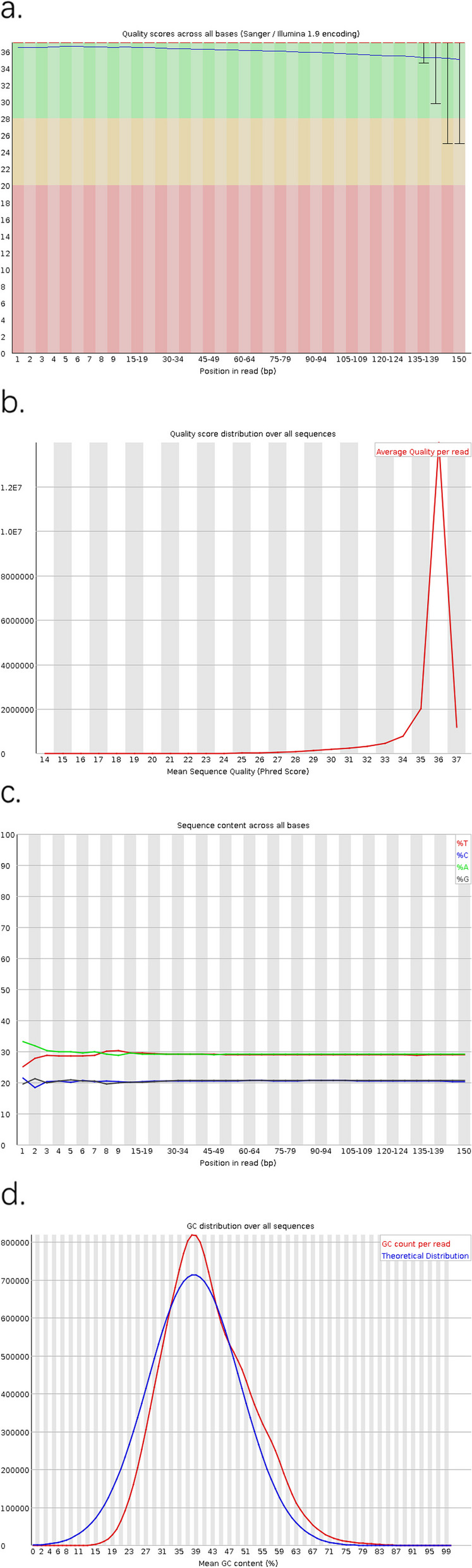
**a** Statistical distribution of sequencing quality for each base locus of all sequences; **b** average distribution of sequencing quality; **c** Distribution diagram of base species at each point of all sequences; **d** Statistical diagram of GC content distribution of sequences

 The mass distribution, average mass and the content of four bases of the original sequencing data showed an average degree at each point, and the GC content of the sequencing data was close to a normal distribution, indicating that the data were reliable. Therefore, the process of sample collection, extraction and sequencing library preparation met expectations, and subsequent analysis could be carried out (Fig. 2a, b, c, d). The number of total and high-quality reads obtained in the DM-DE group were 67691541300 and 66968811965; The number of total and high-quality reads obtained in the DM-NDE group were 66021441600 and 65309847151; The number of total and high-quality reads obtained in the NDM group were 64689658500 and 64038941080. Cutadapt (v1.17) was used to identify potential junction sequences at the 3’ end and cut at the identified junction sequences. Fastp (v0.20.0) was used to screen the quality of the sequences by sliding window method, and the sequences less than 50 bp in length and containing fuzzy bases were removed.

### Composition and difference analysis of the ocular microbiome

**Fig. 3:**
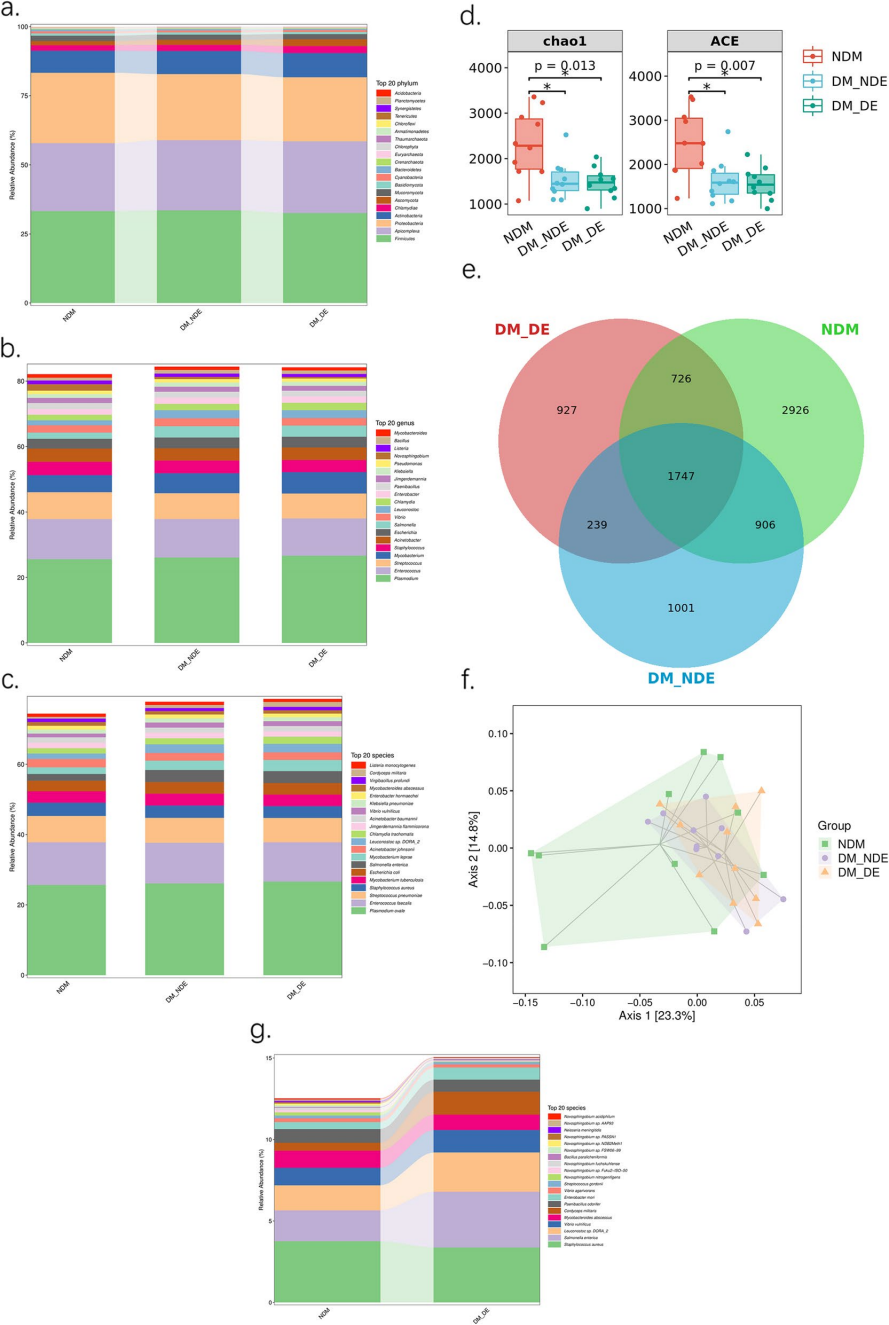
**a** The relative abundance of three groups at the phylum level (top 20); **b** The relative abundance of three groups at the genus level (top 20); **c** The relative abundance of three groups at the species level (top 20); **d** α-diversity index analysis of the three study groups (including Chao1 index and ACE index); **e** Venn diagram analysis of three groups; **f** PCoA of the three study groups; **g** Species composition with significant differences at the species level between the NDM and DM-DE groups (top 20)

 A total of 7261 bacteria, 126 archaea, 980 eukaryotes and 105 viruses were detected in the ocular surface conjunctival swabs of the three groups. Firmicutes, Apicomplexa, Proteobacteria, Actinobacteria, Chlamydiae, Ascomycota, Mucoromycota, Basidiomycota, Cyanobacteria, and Bacteroidetes were the dominant phyla, accounting for more than 90% of the total abundance (Table 2). *Plasmodium, Enterococcus, Streptococcus, Mycobacterium, Staphylococcus, Acinetobacter, Escherichia, Salmonella, Vibrio, Leuconostoc, Chlamydia, Enterobacter, Paenibacillus, Jimgerdemannia, Klebsiella, Pseudomonas, Novosphingobium, Listeria, Bacillus* and *Mycobacteroides* were the dominant genera (more than 1% in abundance). *Plasmodium ovale, Enterococcus faecalis, Streptococcus pneumoniae, S. aureus, Mycobacterium tuberculosis, Escherichia coli, Salmonella enterica, Mycobacterium leprae, Acinetobacter johnsonii, Leuconostoc sp. DORA_2, Chlamydia trachomatis, Jimgerdemannia flammicorona, Acinetobacter baumannii, Vibrio vulnificus, Klebsiella pneumoniae, Enterobacter hormaechei, Mycobacteroides abscessus, Virgibacillus profundi, Cordyceps militaris* and *Listeria monocytogenes* were the dominant species (with an abundance greater than or close to 1%). At the phylum level, the abundance of Apicomplexa, Actinobacteria, Chlamydiae and Ascomycota in the DM-DE group was significantly higher than that in the NDM group. The abundance of Firmicutes and Proteobacteria in the NDM group was significantly higher than that in the DM-DE group. At the genus level, the abundance of *Plasmodium, Mycobacterium, Escherichia, Vibrio, Leuconostoc, Chlamydia* and *Enterobacter* in the DM-DE group was significantly higher than that in the NDM group. The abundance of *Enterococcus, Streptococcus, Staphylococcus* and *Acinetobacter* in the NDM group was significantly higher than that in the DM-DE group. At the species level, the abundance of *P. ovale, E. coli, S. enterica, M. leprae, L. sp. DORA_2* and *C. trachomatis* in the DM-DE group were significantly higher than those in the NDM group. The abundance of *E. faecalis, S. pneumoniae, S. aureus* and *A. johnsonii* in the NDM group was significantly higher than that in the DM-DE group. The abundance of dominant species in the DM-DE group was the highest at the phylum, genus and species levels (Fig. 3a, b, c). The species Venn diagram showed that a total of 3639 microbial species were annotated in the DM-DE group, 3893 microbial species were annotated in the DM-NDE group, and 6305 microbial species were annotated in the NDM group. A total of 1747 microbial species were annotated in the three groups. A total of 927 microbial species were unique to the DM-DE group, 1001 microbial species were unique to the DM-NDE group, and 2926 microorganisms were unique in the NDM group. Additionally, the total number of annotated species and unique species in the DM-DE group were the lowest (Fig. 3d). The alpha diversity results showed the lowest microbial diversity in the DM-DE group and the highest microbial diversity in the NDM group (Fig. 3e). PCoA showed that the beta diversity of microorganisms on the ocular surface was different among the three groups (Adonis test: *R* = 0.12208, *p* = 0.047) (Fig. 3f). LEfSe analysis revealed that *S. aureus, S. enterica, L. sp. DORA_2, V. vulnificus, M. abscessus, Cordyceps militaris, Paenibacillus odorifer, Enterobacter mori, Vibrio agarivorans, Streptococcus gordonii, Novosphingobium nitrogenifigens* and *Novosphingobium sp. Fuku2 − ISO − 50* were significantly different species from the NDM group and the DM-DE group. Among them, *S. aureus, M. abscessus, P. odorifer, V. agarivorans, S. gordonii, N. nitrogenifigens* and *N. sp. Fuku2 − ISO − 50 were clustered in the NDM group, S. enterica, L. sp. DORA_2, V. vulnificus, Cordyceps militaris* and *E. mori* were clustered in the DM-DE group (Fig. 3g).

**Table 2:** Relative species abundance at phylum level of 3 groups (top 20)

Taxon	NDM	DM_NDE	DM_DE
k__Bacteria;p__Firmicutes	33.28%	33.55%	32.59%
k__Eukaryota;p__Apicomplexa	24.56%	25.37%	25.94%
k__Bacteria;p__Proteobacteria	25.48%	23.93%	23.17%
k__Bacteria;p__Actinobacteria	7.97%	8.33%	8.76%
k__Bacteria;p__Chlamydiae	1.98%	2.21%	2.52%
k__Eukaryota;p__Ascomycota	1.52%	1.84%	2.45%
k__Eukaryota;p__Mucoromycota	1.93%	1.87%	1.87%
k__Eukaryota;p__Basidiomycota	0.81%	0.80%	0.74%
k__Bacteria;p__Cyanobacteria	0.75%	0.71%	0.69%
k__Bacteria;p__Bacteroidetes	0.71%	0.55%	0.46%
k__Archaea;p__Crenarchaeota	0.33%	0.31%	0.30%
k__Archaea;p__Euryarchaeota	0.17%	0.18%	0.17%
k__Eukaryota;p__Chlorophyta	0.10%	0.08%	0.09%
k__Archaea;p__Thaumarchaeota	0.04%	0.06%	0.05%
k__Bacteria;p__Armatimonadetes	0.06%	0.02%	0.01%
k__Bacteria;p__Chloroflexi	0.04%	0.01%	0.01%
k__Bacteria;p__Tenericutes	0.01%	0.02%	0.02%
k__Bacteria;p__Synergistetes	0.02%	0.02%	0.02%
k__Bacteria;p__Planctomycetes	0.03%	0.01%	0.01%
k__Bacteria;p__Acidobacteria	0.03%	0.01%	0.01%

### Functional level analysis

#### KEGG [24] metabolic pathway statistics

**Fig. 4:**
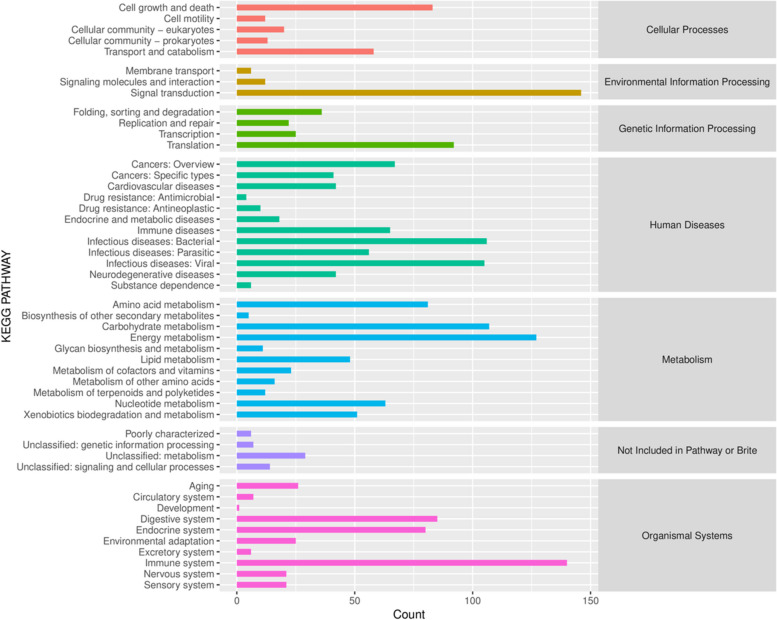
Statistical diagram of KEGG metabolic pathway annotation results

In this study, 15 million nonredundant functional genes were extracted from the shotgun metagenome set. According to the KEGG [24] database, a total of 2099 functional pathways were obtained, which were divided into different KEGG [24] functional pathways. The most commonly annotated functional genes were Human Diseases (26.8%), followed by Metabolism (25.9%), Organismal Systems (19.6%), Cellular Processes (8.9%), Genetic Information Processing (8.3%), Environmental Information Processing (7.8%) and Not Included in Pathway or Brite (2.7%) (Fig. 4).

#### Analysis of functional composition (KEGG [24] metabolic pathway and EggNOG functional groups)

**Fig. 5:**
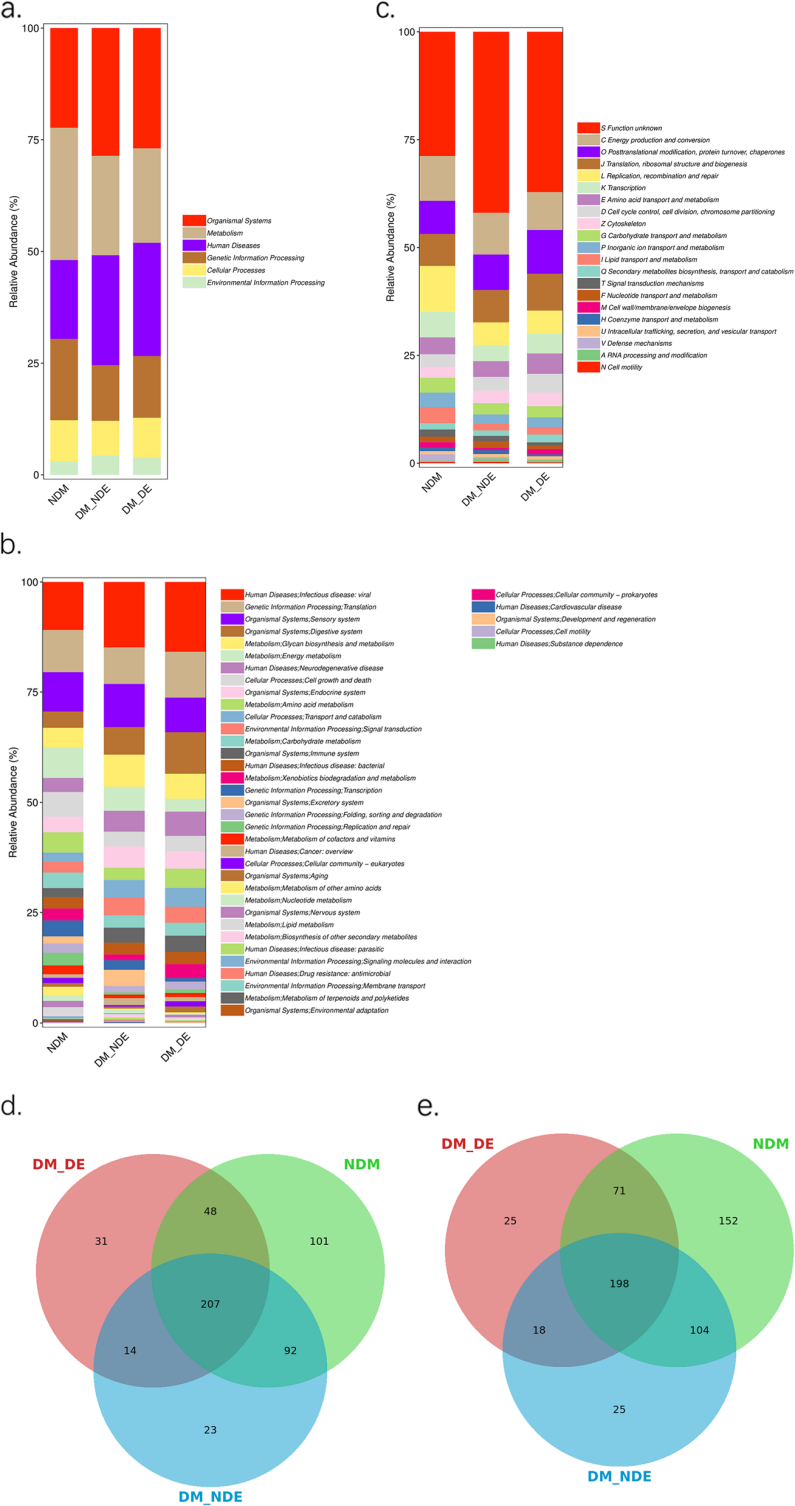
**a** Composition of KEGG primary metabolic pathway in 3 groups; **b** Composition of KEGG secondary metabolic pathway in 3 groups; **c** EggNOG functional group annotation results statistical chart; **d** Venn diagram of KEGG metabolic pathway; **e** Venn diagram of EggNOG functional groups

 The results of the KEGG [24] primary metabolic pathway in each group showed the highest relative abundance of human diseases in the DM-DE group, and the results of the KEGG [24] secondary metabolic pathway in each group showed that infectious disease-bacterial, infectious disease-viral, neurodegenerative disease and substance dependence related to the function of biological metabolic pathways had the highest relative abundance in the DM-DE group, while the relative abundance of human diseases and drug resistance-antimicrobial was the lowest in the DM-DE group, indicating that there were more disease-related genes in the DM-DE group and antibiotic resistance genes in healthy children. EggNOG functional groups showed that genes related to carbohydrate, coenzyme and lipid transport and metabolism were relatively abundant in the DM-DE group, while replication, recombination, repair, signal transduction and defense genes were relatively abundant in the NDM group (Fig. 5a, b, c). The Venn diagram showed that the unique metabolic pathways and functional groups in the DM-DE group were significantly reduced compared with those in the NDM group (Fig. 5d, e).

#### Function difference analysis

**Fig. 6:**
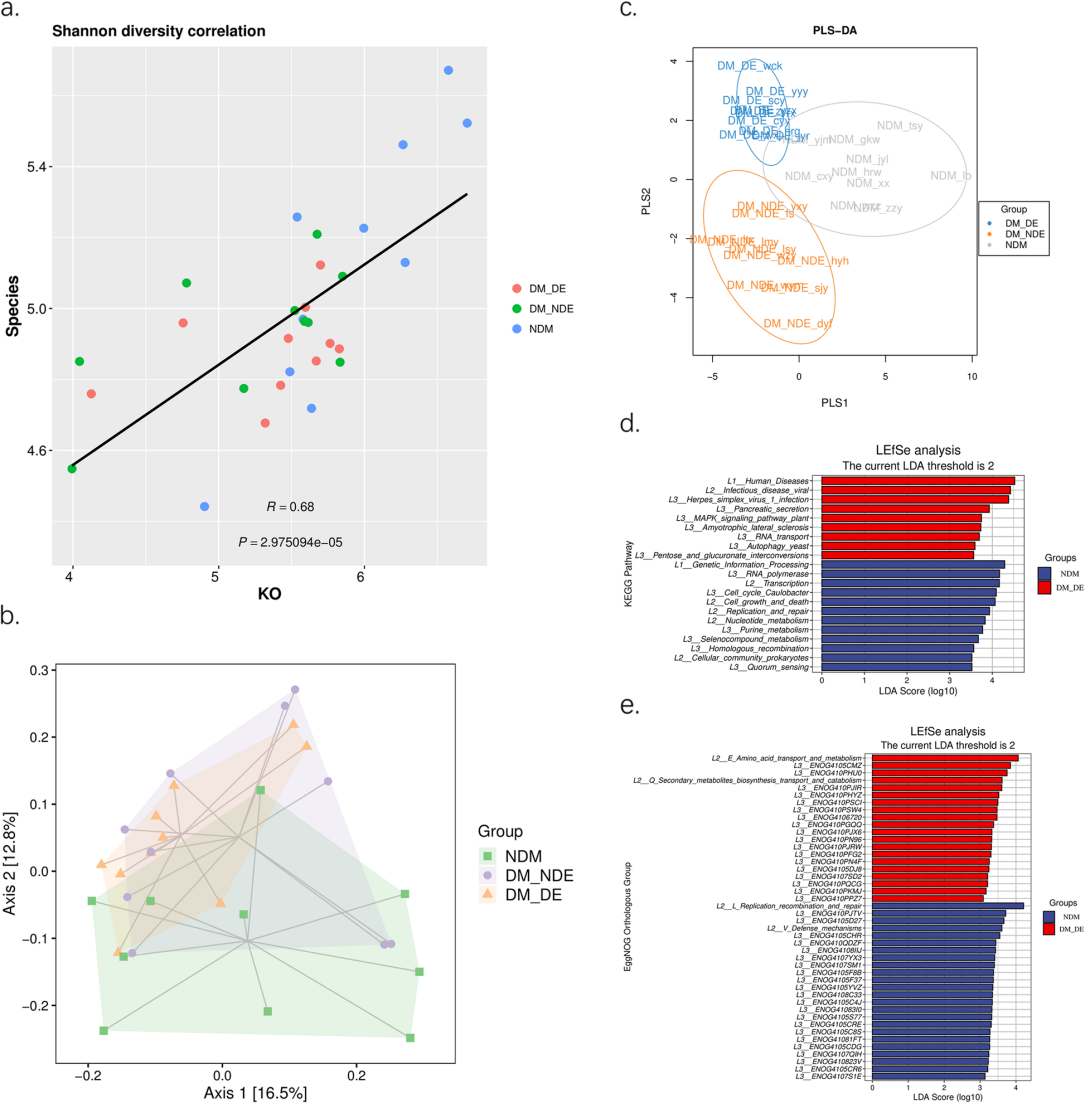
**a** Correlation analysis diagram of Shannon index between species and function; **b** Functional PCoA analysis of the three study groups; **c** Functional composition PLS-DA discriminant analysis diagram; **d** Difference analysis of KEGG metabolic pathway between NDM group and DM-DE group; **e** Difference analysis of EggNOG functional groups between NDM group and DM-DE group

 The Pearson correlation analysis of the Shannon diversity index from both the function level and species level was conducted. The results, which are shown in Fig. 6a (*R* = 0.68, *P* = 2.975094e-05), indicate that there were differences in function and species among all groups. Functional PCoA showed that the beta diversity of microorganisms on the ocular surface was different among the three groups (ANOSIM test: *R* = 0.1367, *p* = 0.006; Adonis test: *R* = 0.10436, *p* = 0.014) (Fig. 6b). The PLS-DA discriminant model was constructed according to the functional composition spectrum and sample grouping data, and the results showed that the functional differences of the microorganisms on the ocular surface were significant among the three groups (ANOSIM test: *R* = 0.1388, *p* = 0.004) (Fig. 6c). The KEGG [24] database counted metabolic pathways in each group (Fig. 6d). LEfSe analysis showed that functional genes related to human disease and endocrine and MAPK signaling pathways were abundant in the DM-DE group, while functional genes related to genetic information processing, proliferation and repair were abundant in the NDM group. The eggNOG functional group (Fig. 6e) showed that metabolism-related functional genes were more abundant in the DM-DE group.

## Discussion

In this study, considering the high prevalence and incidence of dry eye in children and adolescents with diabetes [1, 3], as well as the existing studies on changes of ocular surface microorganisms with dry eye in adults and children and adolescents with diabetes [8, 25]. Therefore, we conducted shotgun metagenomic sequencing for the first time on the ocular surface microbes of diabetic children and normal children and evaluated the influence of changes in the composition and function of ocular surface microbes on diabetic dry eye in children. Similar to diabetic adults, the prevalence of dry eye in diabetic children is significantly higher than that in normal children [1]. This study found significant differences in the microbiome and functional composition of the ocular surface between diabetic dry eye children and normal children. Specifically, the enriched species of diabetic children with dry eye are *S. enterica, L. sp. DORA_2, V. vulnificus, Cordyceps militaris* and *E. mori*, while the species enriched in normal children were *S. aureus, M. abscessus, P.odorifer, V. agarivorans* and *S. gordonii, N. nitrogenifigens*, and *N. sp. Fuku2 − ISO − 50*. The functional pathways of ocular surface microorganisms in diabetic children with dry eyes were mainly derived from human disease, antibiotic resistance genes, carbohydrate, coenzyme and lipid transport and metabolism-related functional genes; In normal children, the functional pathways were mainly derived from replication, recombination, repair, signal transduction and defense-related functional genes. Further study on the microflora of ocular surface can accurately analyze the role of microflora in the occurrence and development of ocular diseases, and can also be used to guide more accurate drug treatment to adjust the microecological balance and rebuild the microecological environment conducive to the health of ocular surface, so as to achieve the purpose of treating ocular diseases. To date, no relevant reports have been published.

In this study, compared with normal adolescents and children, the DM-DE group had significant differences in the α diversity and β diversity of the OSM, and the α diversity and β diversity were lower than those of normal children. This is consistent with the results reported by Li [9], who conducted 16Sr RNA sequencing analysis on 35 dry eye and 54 non-dry eye subjects and found that the α diversity and β diversity of bacteria in the dry eye group were significantly reduced. This is also consistent with the results reported by Liang [26], who used shotgun metagenomic sequencing on 47 dry eye patients and 48 healthy subjects and found that the α diversity and β diversity of bacteria in the dry eye group were significantly reduced. However, it is contrary to the conclusion of other studies that the α diversity of OSM in DE patients without DM were higher than that in the normal population [9, 11, 15, 27] and contrary to the conclusion of the previous study of our project team that the α diversity and β diversity of OSM in diabetic adults with dry eyes [8] were higher than those in the normal control group. This may be due to the differences in the study population, age, sampling and detection methods. Previous studies have shown that there are differences in ocular surface microbial composition between diabetic people and normal people [8]. With age, the composition and species of microorganisms on the ocular surface will change [28]. The sampling site, wiping depth, degree of force and detection method selected all affect the research results of ocular surface microorganisms to a certain extent [29–31]. In this study, we found that the microflora at the level of dominant phylum, genus and species had the highest abundance in the DM-DE group and the lowest in the NDM group, suggesting that the increased abundance of dominant microflora on the ocular surface may be the cause of dry eye in diabetic children. Increased microbial populations are often harmful to the body because disruption of the homeostasis of the core microbiome of the ocular surface is more likely to lead to disease. This finding suggests that the pathogenesis of dry eye in children may be caused by the destruction of microbial-oriented homeostasis in the host: the changes in the ocular surface of dry eye may lead to changes in the microecology, or the changes in the abundance of microorganisms may lead to dry eye, but the specific mechanism remains unclear.

In this metagenomic examination, we found some special microorganisms in addition to bacteria on the ocular surface. In addition to the main phyla composed of Proteobacteria, Firmicutes and Actinomycetes, Apicomplexa and Chlamydia were also found to be important microbial phyla in the ocular surface, with more species than 16Sr RNA gene sequencing. Additionally, our study found that *Plasmodium ovale, Mycobacterium leprae* and *Chlamydia trachomatis* were highly abundant in the eyes of children with diabetes, especially in children with dry eyes, which had not been reported before. The specific mechanism of these microorganisms in the eyes still needs further confirmation. This was inconsistent with the report by Li [9] that *Bacteroides* and *Bacteroides fragilis* were obviously enriched in the dry eye group, while *Pseudomonas* was mainly enriched in the non-dry eye group. This finding likely due to the difference between the subjects in each study. Our study found that the core ocular microorganisms *Enterococcus faecalis, Streptococcus pneumoniae, S. aureus, Acinetobacter Johnson* and *Acinetobacter baumannii* had the highest abundance on the ocular surface of normal children, and these bacteria may be involved in the causes of dry eye in adults, as previously reported [14, 15]. This difference in results may indicate that the decrease in such microflora on the ocular surface of diabetic children affects the homeostasis of normal microorganisms in the eye and leads to the onset of dry eye in diabetic children.

An important finding of this study was that through the analysis of functional genes, the unique metabolic pathways and functional groups in the DM-DE group were reduced, which was consistent with the decrease in microbial diversity in the ocular surface. The inflammatory response caused by the high glucose environment in the ocular surface inhibits the growth of some microorganisms, leading to a reduction in the metabolic requirements of these microbial substances. Additionally, the increase in viral and bacterial functional genes related to infectious diseases enhanced the production of inflammatory factors and virulence, and the microenvironment of ocular surface homeostasis was destroyed, leading to dry eye. Our study also found that in the DM-DE group, functional genes related to the transport and metabolism of carbohydrates, enzymes and lipids were reduced, which would lead to abnormal metabolism and transport of substances on the ocular surface, thereby damaging the normal components of tears and leading to dry eyes. Compared with the NDM group, the DM-DE group had fewer functional genes related to replication, recombination, repair, signal transduction and defense, and the normal cell metabolism of the ocular surface was inhibited. This abnormality may also be the cause of diabetic dry eye in children, but the specific mechanism needs to be further confirmed. In 2017, Chen Hong [32] et al. conducted metagenomic sequencing analysis on conjunctiva imprinted cell samples from patients with dry eye and found that patients with dry eye were significantly more enriched with antibiotic resistance genes than healthy subjects, which may be related to drug use in patients with dry eye. This was contrary to our findings that the antibiotic resistance gene of diabetic dry eye children was lower than that of normal children. It was speculated that all the subjects tested in our study were children. Even if they had dry eye symptoms, they mostly avoided using eye drops for treatment because of their strong tolerance. Our study also found that the relative abundance of neurodegenerative disease genes in the DM-DE group was the highest, which is consistent with the pathogenesis of dry eye in diabetic children, which is prone to peripheral ocular surface neuropathy, resulting in abnormal blinking, reduced glandular secretion, and accelerated tear evaporation [16, 33].

This study had some limitations. First, due to the impact of the COVID-19 epidemic, the number of children with diabetes who were able to participate in screening for eye diseases was small, resulting in a small sample size. Second, this is the first study to analyze the microbial composition and function of the ocular surface through metagenomic sequencing, as no previous studies have used this approach. Third, due to the low microbial content on the ocular surface and because shotgun metagenomic sequencing removed the influence of host factors, the microbial quantity obtained was lowered. Fourth, metagenomes require a high-quality sample and cannot determine the expression of microorganisms. Fifth, metagenomes require higher sequence coverage than 16Sr DNA sequence analysis, and although the sampling process was performed carefully, host contamination was inevitable.

## Conclusions

In summary, this study found that dry eye in children with DM was accompanied by characteristic changes in OSM and metabolic pathways. The microbial composition and structure of the ocular surface in patients with dry eye were changed, and the change in microflora was an important factor affecting dry eye. The role of microorganisms in dry eye is complex, and more and larger sample studies are needed to clarify the role of microorganisms in the course of dry eye disease and the pathogenic mechanism. In the future, multicenter clinical studies can be carried out to expand the sample size. Microorganisms and functional genes closely related to the development of DM-DE can be screened to further explore the pathogenesis of DM-DE in children.

## Data Availability

The detail data and materials in the current study are available from the SRA database under the number PRJNA1010661 or use the following links (https://www.ncbi.nlm.nih.gov/sra/PRJNA1010661).
